# A Heat-Killed Probiotic Mixture Regulates Immune T Cells Balance and IgE Production in House Dust Mite Extraction-Induced Atopic Dermatitis Mice

**DOI:** 10.3390/microorganisms10101881

**Published:** 2022-09-21

**Authors:** Hsin-Yu Chen, Yung-Tsung Chen, Kuan-Yi Li, Hsiao-Wen Huang, Yu-Chun Lin, Ming-Ju Chen

**Affiliations:** 1 Department of Animal Science and Technology, National Taiwan University, Taipei 10617, Taiwan; 2 Department of Food Science, National Taiwan Ocean University, Keelung 202301, Taiwan; 3 Livestock Research Institute, Council of Agriculture, Executive Yuan, Tainan City 712009, Taiwan

**Keywords:** probiotics, atopic dermatitis, immune T cells, immunoglobulin E

## Abstract

Atopic dermatitis (AD) is a chronic and relapsing inflammatory skin disease accompanied with severe itching and skin lesions. Current studies have demonstrated that probiotics can exert an immunomodulatory effect, improve epithelial barrier function, and normalize the composition of gut microbiota. Thus, the aim of this study was to investigate the effect of probiotics on the immune balance of AD in vivo. We first screened two lactic acid bacteria strains, which were *Lactococcus lactis* subsp. *cremoris* MP01 and *Lactobacillus paracasei* subsp. *paracasei* MP02, from 10 strains isolated from traditional fermented milk with inflammation regulating activities in vitro. In the house dust mite (HDM) extraction-induced AD mouse model, mice were assigned randomly to four groups: control group (PC), HDM-induced AD group (NC), HDM-induced AD mice with administration of a mixture of heat-killed MP01 and MP02 at a low concentration (LD), and high concentration (HD) groups. Compared with the NC group, the probiotic treatments could relieve the AD symptoms. Moreover, the LD group significantly decreased total and HDM-specific IgE concentration. These results indicated that a combination of heat-killed MP01 and MP02 strains modulated the proportion of IL4^+^CD4^+^ T cells and IFNγ^+^CD4^+^ T cells in the spleen of HDM extraction-induced AD mice. In conclusion, administration of the heat-killed MP01 and MP02 mixtures appeared to relieve the classic AD signs, decrease serum IgE concentration, and rebalance the population of Th1/Th2 cells in HDM extraction-induced AD mice. The immunomodulatory activities of a combination of heat-killed MP01 and MP02 provided a potential new therapeutic strategy against AD.

## 1. Introduction

Atopic dermatitis (AD), a chronic inflammatory skin disorder, is a complex disease with the symptoms, including red, dry, scaly, and itchy skin. AD is often diagnosed in young children, and less commonly in adults worldwide [1]. Genetic disfunction [2] and abuse of antibiotics [3] were recently recognized as the predisposing factors to the development of AD. In addition, the imbalance of the immune response is one of characteristics of AD. The Th2/Th17 immune response and eosinophil infiltration in the skin have been demonstrated to dominate the early stage of AD, while Th1/Treg is a dominant mediator in the chronic phase of AD [4]. Several clinical interventions have been developed to attenuate the itch–scratch cycle, skin inflammation, or improve epithelial barrier impairment in AD. The medicines such as corticosteroids and calcineurin inhibitors are widely considered as effective clinical therapies for AD but can cause side effects [5]. Therefore, an alternative therapy for AD treatment needs to be discovered.

Changes in gut microbial composition were recently associated with the development of AD [6]. Accumulating evidence has demonstrated that probiotics had the ability to modulate the immune response [7] and manipulate gut microbiota [8] which could result in the improvement of AD. A previous study showed that Lactobacillus GG supplementation significantly attenuated the clinical score of AD in infants [9]. A specific probiotic mixture particularly improving AD in children with a skin prick test response and increasing IgE level had been demonstrated [10]. Moreover, the intervention of a heat-killed Lactobacillus strain showed the positive effects on house-dust-mite-induced atopic dermatitis in NC/Nga mice [11]. However, the efficacy of probiotic treatment in AD should be considered when the number of subjects was limited and diagnostic criteria of AD was unclear in the clinical trial. Therefore, the detailed mechanisms of improving AD by using a probiotic treatment still need to be uncovered.

In the present study, we screened the potential strains based on the level of secretory inflammatory cytokines TNF-α and IL-10 in murine macrophages cells RAW264.7 cocultured with 10 different Lactobacillus strains isolated from fermented milk. To reduce the risk of opportunistic infection caused by viable probiotics in the population groups with defective immunity [12], the heat-killed bacteria strains were used in the present study. Furthermore, this study evaluated the oral administration of heat-killed selected strains on the regulation of immune response in house dust mite extract (HDM)-induced AD mice.

## 2. Materials and Methods

### 2.1. Bacteria Preparation

Lactic acid bacteria were isolated from traditional fermented milk. Each strain was cultured two times before experiments. After bacterial activation, candidate strains were washed with phosphate-buffered saline solution (Uni Biotech Co., Ltd., Yesan-Gun, Korea) and suspended in Dulbecco’s Modified Eagle’s Medium (DMEM, Corning Inc., Corning, NY, USA) with 10% (*v/v*) heated fetal bovine serum (FBS, Thermo Fisher Scientific Inc., Waltham, MA, USA) for further in vitro experiments. On the other hand, the bacterial suspensions for mouse experiments were prepared weekly. In brief, the strains were washed with PBS and suspended in PBS with concentrations of 10^7^ and 10^9^ CFU/mL, respectively, and stored at 4°C. The prepared bacterial suspensions were heated at 85°C for 40 min before feeding.

For identification of LAB, the 16S rRNA gene was amplified by using primers 8F (5′-AGAGTTTGATCMTGGCTCAG-3′) and 15R (5′-AAGGAGGTGATCCARCCGCA-3′). A 25 μL PCR reaction solution included 10× Taq PCR buffer (Takara Bio Inc., Kusatsu, Shiga, Japan), 0.2 mM dNTP mixture solution (Takara Bio Inc.), 1 U/μL Taq DNA polymerase (Takara Bio Inc.), 0.8 μM primer 8F, 0.8 μM primer 15R, and 100 ng DNA template. The PCR conditions were as follows: 94 °C for 5 min, 35 cycles of 94 °C for 30 s, 58 °C for 30 s, and 72 °C for 90 s, followed by 72 °C for 10 min. The 16S rRNA gene sequences were analyzed by using Basic Local Alignment Search Tool (BLAST) at the National Center for Biotechnology Information (NCBI) database to discriminate bacteria at the species level.

### 2.2. Anti-Allergic Potential Strains Screening Strategy

The mouse macrophage cell line RAW cell 264.7 was purchased from Bioresource Collection and Research Center, Food Industry Research and Development Institute (Hsinchu, Taiwan), and cultured in DMEM with 10% (*v/v*) heated FBS and 1% Antibiotic-Antimycotic solution (Corning Inc.) at 5% CO_2_ and 37 °C. For the strain-screening strategy, cells were suspended in DMEM with 10% (*v/v*) heated FBS and cultured in 24-well plates (4 × 10^5^ cells/mL) under the same incubating conditions for 24 h. Each well was washed gently with Dulbecco’s Phosphate-Buffered Saline (DPBS, Thermo Fisher Scientific Inc.) and grouped into different treatments: DMEM with 10% (*v/v*) heated FBS (Negative Control, NC), 50 ng/mL lipopolysaccharide (Positive Control, PC), and DMEM with 10% (*v/v*) heated FBS and different candidate strains (4 × 10^4^ CFU/mL). Cells and strains were co-cultured under the same incubating conditions for 24 h. Later, the supernatants were collected and stored at −20 °C for further cytokine measurements. The concentrations of tumor necrosis factor-alpha (TNF-α) and interleukin (IL)-10 were measured with mouse cytokines ELISA kits (R&D Systems, Inc., Mckinley, MN, USA). All procedures followed the instruction manuals.

### 2.3. Animals

Five-week-old male BABL/c mice were purchased from BioLasco Taiwan Co., Ltd. and housed at the National Taiwan University (NTU) Animal Resource Center. The environment was maintained on a 12 h light/dark cycle at 20 ± 1°C with food and water ad libitum. After a 1-week acclimatization, the mice were randomized by body weight into four groups: (1) health mice (positive control, PC), (2) AD-induced mice (negative control, NC), (3) AD-induced mice with low dosage bacterial treatment (1 × 10^7^ CFU/mL, 200µL per mouse, LD Group), and (4) AD-induced mice with high-dosage bacterial treatment (1 × 10^9^ CFU/mL, 200 µL per mouse, HD Group). The dosage used in this study was developed with reference to a previous report [13]. The experimental procedure was in accordance with the 2018 Guideline for the Care and Use of Laboratory Animals (Taiwan). The changes in body weight and behavior of the test mice were observed. The study protocol was approved by the Institutional Animal Care and Use Committee (IACUC) at NTU (Approval No. NTU-107-EL-00054).

### 2.4. Sensitization and Bacterial Treatment

AD-like lesions induced by HDM extraction in BALB/c mice was performed as previously described [13,14] with modification. In brief, the mice were sensitized intraperitoneally with 20 μg HDM extraction (Stallergenes Greer, Lenoir, NC, USA) and 2 mg aluminum hydroxide hydrate (Sigma-Aldrich Inc., Burlington, MA, USA) in 200 μL PBS on Day 13, Day 20, and Day 27. The dorsal sides of mice were shaved with an electric razor and hair removal cream (Church & Dwight Co., Inc., Ewing, NJ, USA) on Day 33. Subsequently, the mice were challenged by the sterile gauzes with 50 μL HDM extraction in PBS (2 mg/mL) on the dorsal side three times. Finally, the mice were sacrificed with serum, dorsal skin, spleen, mesenteric lymph nodes (MLN), and Peyer’s Patches (PP) collected for further analysis on Day 46. The placebo and bacterial treatments were provided to mice by oral gavage every day during the experimental period.

### 2.5. Histological Section

The dorsal skin of mice was collected and fixed in formaldehyde solution, 10% (*w/v*) in aqueous phosphate buffer (Mallinckrodt backer, Inc., Phillipsburg, NJ, USA) for 24 h. The preparation of tissue slides and staining processes were conducted by Raya Biotech Ltd., Taipei, Taiwan. In brief, the samples were fixed with paraffin sections and stained with hematoxylin and eosin stain (H&E). Subsequently, the slides were evaluated and analyzed by the Animal Disease Diagnostic Center, National Chung-Hsing University (NCHU). The severity of four symptoms (hyperplasia, hyperkeratosis, inflammation, and ulceration) was evaluated using the percentage of symptoms compared to the whole slice area. The degree of lesions was graded from 1 to 5 depending on severity: 1 (minimal, <1%), 2 (slight, 1–25%), 3 (moderate, 26–50%), 4 (moderate/severe, 51–75%), and 5 (severe/high, 76–100%). Moreover, the thickness of the epidermis and dermis was examined by selecting three different microscopic high-power fields (HPF, 400×) per slide. Two slides per mouse were measured. The mean of the measurements represents the thickness per mouse.

### 2.6. Total Serum IgE Analysis

The mouse whole-blood samples were taken using orbital sinus blood collection and centrifuged at 4 °C (2000× *g*, 5 min). The serum samples were collected and stored at −80 °C for further analysis. Serum total IgE concentration was measured using the Mouse IgE Uncoated ELISA kit (Thermo Fisher Scientific Inc.), while HDM-specific IgE concentration was quantified using the Mouse Anti-House Dust Mite (HDM) IgE Antibody Assay Kit (Chondrex, Inc., Redmond, WA, USA). All procedures followed the instruction manuals.

### 2.7. Cytokine Production in Mesenteric Lymph Nodes (MLN)

The tissue protein was extracted using the T-PER Tissue Protein Extraction Reagent (Thermo Fisher Scientific Inc.). The concentrations of cytokines (IL-2, IL-4, IL-6, IFN-γ, TNF, IL-17A, and IL-10) were quantitated with the BD Cytometric Bead Array (CBA) Cytokine Kit (BD Biosciences) with a flow cytometer (BD FACSCanto™ II, BD Biosciences).

### 2.8. The Measurement of T Cell Staining

The primary cells were isolated from Peyer’s Patches (PP) and spleens. The Treg cells in PP were stained by BB515 rat anti-mouse CD25 antibody, APC rat anti-mouse CD4 antibody, and PE Rat anti-mouse Foxp3 antibody (BD Biosciences). The Th1/Th2/Th17 cells in the spleen were stained with a Mouse Th1/Th2/Th17 Phenotyping Kit (BD Biosciences). Later, the stained cells were analyzed using a flow cytometer. All procedures followed the instruction manuals.

### 2.9. Statistical Analysis

The statistical analyses were performed using Statistical Analysis System (SAS) v9.4 and Prism v6.0 (GraphPad Software Inc., San Diego, CA, USA). The results of in vitro experiments were analyzed by using ANOVA with Duncan’s multiple comparison tests. The other results were analyzed by using ANOVA with Tukey’s multiple comparison test or Dunnett’s multiple comparison tests. The values were described as mean ± SD, and the *p* value of <0.05 was considered significant.

## 3. Results

### 3.1. Effects of Lactobacillus Strains Isolated from Fermented Milk on Inflammatory Cytokine Secretion In Vitro

**Figure 1:**
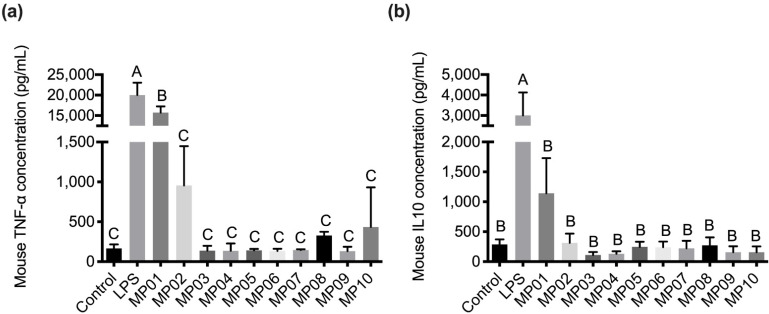
Effects of the different probiotics on the production of TNF-α (**a**) and IL10 (**b**) after 24 h co-culture with RAW 264.7. NC: negative control; PC: positive control (50 ng/mL LPS). Data were analyzed by ANOVA with Duncan’s multiple comparison tests. Means for each group without a common letter are significantly different (*p* < 0.05). Value represents means ± SD (n = 3).

To screen the potential strains inducing an immune response in vitro, the levels of inflammatory cytokines TNF-α and IL-10 in murine macrophages RAW264.7 cells co-cultured with 10 Lactobacillus strains from fermented milk were examined. The results showed that no cytotoxicity on RAW264.7 cells was found in all strains at 4 × 10^5^ CFU with the MTT assay (Supplementary Figure S1). LPS treatment significantly induced TNF-α and IL-10 cytokines production in RAW264.7 cells (Figure 1). However, a low level of TNF-α and IL-10 cytokines production was detected in all test strains except MP01 and MP02. The MP01 strain induced the highest production of TNF-α and IL-10 cytokines in RAW264.7 cells. Additionally, the MP02 strain slightly induced TNF-α secretion. Therefore, MP01 and MP02 were chosen as the most effective strains for conducting the in vivo study. Furthermore, MP01 and MP02 were identified as *Lactococcus lactis* subsp. *cremoris* and *Lactobacillus paracasei* subsp. *paracasei* by 16S rRNA gene sequencing (Supplementary Table S1).

### 3.2. A Mixture of Heat-Killed MP01 and MP02 Strains Alleviated Skin Lesions in HDM Extraction-Induced AD Mice

**Figure 2:**
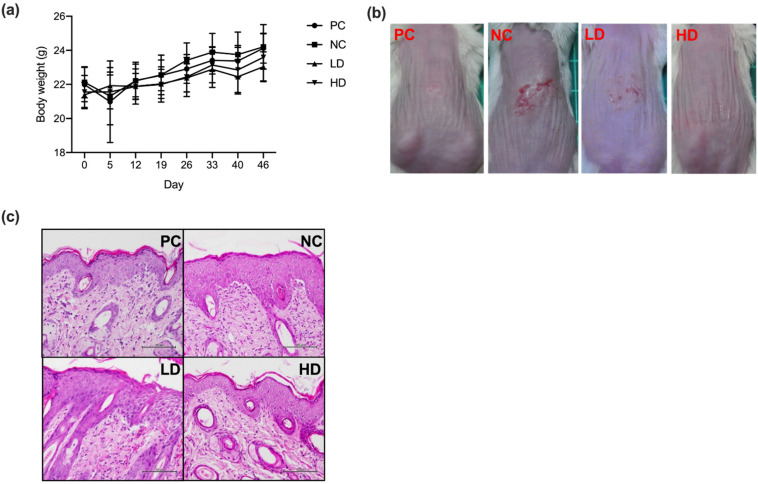
A combination of heat-killed MP01 and MP02 strains alleviated skin lesions of HDM-extraction induced AD mice. (**a**) The body weight of mice after treatment for 46 days. Value represents means ± SD. (n = 7–10/group) (**b**) The skin lesion induced by the HDM extraction in NC, PC, LD, and HD groups. Aspects of dermatitis in AD mice were examined on day 45. (**c**) Histological section of skin of the HDM-induced AD in NC, PC, LD, and HD groups. The dorsal skin from mice was stained by hematoxylin and eosin stain, and the slides were examined under 400× magnification.

To evaluate the therapeutic effect of a mixture of heat-killed MP01 and MP02 on atopic dermatitis, AD-like lesions induced by HDM extraction in BALB/c mice were examined. The histological section of the skin from HDM-extraction-induced AD mice were analyzed. The results showed that all treatments had no adverse effects on body weight of test mice in groups during the experimental period (Figure 2a). After 45 days of the treatment, compared with the PC group, all the other groups, including NC, LD, and HD groups, showed the dermatitis symptoms, such as redness, abrasion, and crusting. The NC group developed severe dermatitis after the induction of HDM extraction (Figure 2b). Both LD and HD groups had slightly reduced dermatitis symptoms in comparation with the NC group. The results of skin histological sections showed that the HDM extraction induced an increase in epidermal thickening in the NC group (Figure 2c). By contrast, LD and HD groups showed a trend of improvement in epidermal hyperplasia compared with the NC group. These results showed that a combination of heat-killed MP01 and MP02 ameliorated skin lesions and inflammatory cell infiltration induced by HDM extraction in mice.

### 3.3. A Mixture of Heat-Killed MP01 and MP02 Strains Regulated the Number of Th1 and Th2 Cells in the Spleen of HDM-Extraction-Induced AD Mice

**Figure 3:**
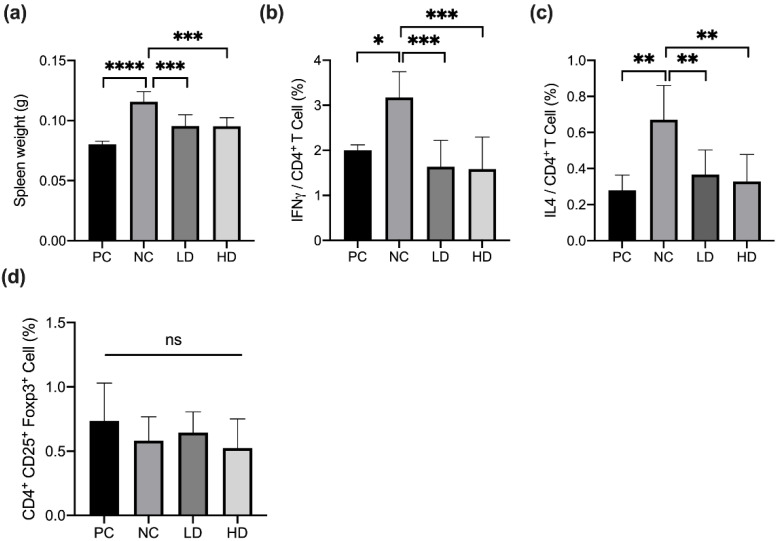
A combination of heat-killed MP01 and MP02 strains rebalanced the proportion of Th1 and Th2 cells in the spleen of HDM-extraction-induced AD mice. (**a**) The spleen weight of mice after treatment for 46 days. The population of (**b**) IFN-γ^+^CD4^+^ T cells, (**c**) IL4^+^CD4^+^ T cells, and (**d**) CD4^+^CD25^+^Foxp3^+^ T cells in the spleen of test mice. The columns and the error bars represent means ± SD (n = 7–10/group). Data were analyzed using a one-way ANOVA with Tukey’s multiple comparison test. * *p* < 0.05, ** *p* < 0.01, *** *p* < 0.001. Value represents means ± SD.

The T-helper cell differentiation involved in the progression of atopic dermatitis had been demonstrated [4]. To determine the effect of a mixture of heat-killed MP01 and MP02 on systemic T cell responses, the weight of spleen and the population of IL4^+^CD4^+^ T cells, IFN-γ^+^CD4^+^ T cells in the spleen, and CD4^+^CD25^+^Foxp3^+^ T cells in the Peyer’s patch of HDM-extraction induced AD mice using flow cytometry are shown in Figure 3. The results showed that the spleen weight of the NC group was significantly increased compared with that of the PC group (Figure 3a). In addition, HDM extraction significantly induced an increase in the population of IL4^+^CD4^+^ T cells and IFN-γ^+^CD4^+^ T cells in the spleen of test mice compared to that of the PC group (Figure 3b,c). 

After treatment for 46 days, both LD and HD groups had significantly reduced spleen weights and the population of IL4^+^CD4^+^ T cells and IFN-γ^+^CD4^+^ T cells in the spleen in comparison with that of the NC group (Figure 3b,c). However, no changes were found in the proportion of CD4^+^CD25^+^Foxp3^+^ T cells in the Peyer’s patch among all groups (Figure 3d). These results indicated that a combination of heat-killed MP01 and MP02 strains modulated the proportion of Th1- and Th2-type T cells in the spleen of HDM-extraction-induced AD mice. Furthermore, to examine allergen-specific immune responses, the levels of TNF-α, IFN-γ, IL-10, and IL-2 in mesenteric lymph nodes and IL-10 and TNF-α cytokines in the serum of HDM-extraction-induced AD mice after treatment was measured (Supplementary Figure S2). The results showed that the NC group slightly reduced the production of IFN-γ and IL-10 and increased IL-2 cytokines in mesenteric lymph nodes of mice in comparison with the PC group. In contrast, the LD group slightly increased the level of IL-10 cytokines in mesenteric lymph nodes compared with the NC group, but there was no significant difference.

### 3.4. A Mixture of Heat-Killed MP01 and MP02 Strains Decreased the Level of HDM-Specific IgE in HDM-Extraction-Induced AD Mice

**Figure 4:**
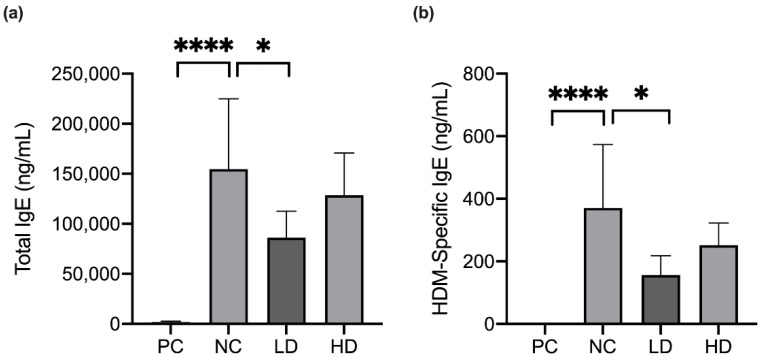
A combination of heat-killed MP01 and MP02 strains decreased the level of HDM-specific IgE (**a**,**b**) in HDM-extraction induced AD mice. (n = 7–10/group) Data were analyzed using an ANOVA with Dunnett’s multiple comparison tests. * *p* < 0.05, **** *p* < 0.0001. Value represents means ± SD.

To evaluate the immune response in the pathogenic inflammation, the effects of a combination of heat-killed MP01 and MP02 strains on the total IgE and HDM-specific IgE in the serum were determined (Figure 4). In the NC group, HDM extraction significantly induced an increasing level of total IgE and HDM-specific IgE in the serum when compared with the PC group. 

By contrast, the LD group significantly downregulated the production of total IgE and HDM-specific IgE levels in the serum. However, the HD group did not modulate the IgE level in the serum of HDM-extraction-induced AD mice.

## 4. Discussion

In recent years, environmental hygiene has attracted attention to prevent allergic reactions and to control allergies. House dust mites have been recognized as the highly relevant allergen for the development of atopic dermatitis [15]. The prevalence of atopic dermatitis, a chronic inflammatory skin disease, is continually increasing worldwide in young children especially [1]. In the present study, we first screened the potential strains from fermented milk based on the cytotoxicity and the ability to induce immune cytokines in macrophage RAW264.7 cells. As a result, MP01 and MP02, which were identified as Lactococcus lactis subsp. cremoris and Lactobacillus paracasei subsp. paracasei with 16S rRNA gene sequencing, respectively, as effective stains by inducing TNF-α and IL-10 cytokine production was demonstrated. We further observed that a combination of heat-killed MP01 and MP02 strains not only ameliorated skin lesions but also regulated the proportion of Th1 and Th2 cells in the spleen and decreased the level of HDM-specific IgE in HDM-extraction-induced AD mice.

Accumulating evidence has demonstrated that intestinal microorganisms and allergens interact with a large number of immune cells residing in the intestine of humans and animals. The toll-like receptor of macrophages distinguishes the antigens from microorganisms, followed by a consequential immune response to balance the immune stability [16,17]. Probiotics have demonstrated multiple health benefits, including immune modulation. When co-cultured with the mouse macrophage cell line RAW 264.7, *Lactobacillus rhamnosus* [18] and *Lactobacillus kefiranofaciens* [19] strains had the ability to stimulate the production of TNF-α and IL-10 cytokines in culture medium. IL-10 has been identified as a regulatory cytokine with the property of immune regulation that prevents pathogen-induced inflammation, thereby maintaining immune homeostasis in the host [20]. In the present study, the probiotic strains were selected by determining cytokine production after co-culture with the mouse macrophage cell line RAW 264.7 for 24 h. The results indicated that the survival rate of RAW 264.7 remained at above 80%, showing that the test strains at 4 × 10^5^ CFU/mL concentrations did not cause cytotoxicity on cells. Moreover, MP01 and MP02 strains effectively induced TNF-α and IL-10 cytokine production in RAW 264.7 cells, suggesting that MP01 and MP02 strains could promote macrophage activation and had immunomodulatory ability in vitro.

The level of serum IgE is the most important indicator in atopic dermatitis (AD) [21,22]. Allergens from food and microbes stimulated Langerhans cells, and consequently induced Th2 immune responses, which promoted IgE-producing plasma cell differentiation in the skin. The IgE is involved in the production of inflammatory cytokines, including IL-4, IL-5, and TNF-α, causing an allergic inflammatory response in the progression of AD [23]. In addition, the heat-killed probiotic strain that downregulated total IgE production in atopic dermatitis NC/Nga mice had been reported [24]. In this study, we observed similar results that the mixture of heat-killed MP01 and MP02 significantly reduced serum total IgE and HDM-specific IgE with the possible mechanisms including repressing the population of Th2 T cells in HDM extraction-induced AD mice, however, it should be clarified in future studies.

Modulating the Th cell immune response had been proposed as therapy for allergic and autoimmune disorders [25]. Previous studies indicated that inhibiting the Th2-dominated inflammation could be an efficient strategy for AD treatment [26]. Recently, it has been shown that the Th1/Th2 and Th17/Treg cells are associated with different stages in the pathogenesis of AD. Th2 and Th17 immune responses mainly dominate the acute phase of AD, while Th1 and Treg responses are involved in the later chronic progression of AD [4]. The administration of probiotics regulating the Th2/Th1 immune response had been studied in the prevention and treatment of allergic diseases [27]. Moreover, a probiotic mixture had demonstrated the ability to upregulate the Th1-mediated response as well as suppress Th2 and Th17 responses in AD-like mice [28]. However, our results demonstrated that the mixture of heat-killed MP01 and MP02 strains reduced IL4^+^CD4^+^ T cells (Th1) and IFNγ^+^CD4^+^ T cells (Th2) cells in the spleen, whereas CD4^+^CD25^+^Foxp3^+^ cells (Treg) in the Peyer’s patch were not changed in HDM-extraction-induced AD mice. This observation indicated that improvement in AD-like skin lesions by treatment with heat-killed MP01 and MP02 strains may be a result of the suppression of both acute and chronic phases in the progression of AD. Further research is needed to investigate the changes in the immune response in different stages of AD with the treatment with heat-killed MP01 and MP02.

## 5. Conclusions

In conclusion, the administration of a mixture of heat-killed MP01 and MP02 significantly relieved the symptoms of AD, decreased serum total IgE and HDM-specific IgE concentration, and rebalanced the population of Th1/Th2 cells in HDM-extraction-induced AD mice. Future studies are necessary to investigate the possible mechanism and the clinical efficacy of these findings should be further evaluated.

## Data Availability

Not applicable.

## Supplementary Materials

The following supporting information can be downloaded at: https://www.mdpi.com/article/10.3390/microorganisms10101881/s1, Figure S1: Effects of the different LAB strains on the cell viability analyzed by MTT assay after 24-h co-culturing with RAW 264.7. NC: Negative control; PC: Positive control (50 ng/mL LPS). Value represents means ± SD. (n = 3). Data were analyzed by ANOVA with Duncan’s multiple comparison tests. Means for each group without a common letter are significantly different (*p* < 0.05); Figure S2: Effects of a combination of heat-killed MP01 and MP02 strains on cytokines production in mesenteric lymph nodes (MLN) and serum of HDM-extraction induced AD mice. (A) TNF-α, (B) IFN-γ, (C) IL-10 and (D) IL-2 in mesenteric lymph nodes and (E) IL-10 and (F) TNF-α cytokines in serum of HDM-extraction induced AD mice after treatment. Data were analyzed by one-way ANOVA with Tukey’s multiple comparison test. Bars indicate means ± SD. Table S1: 16S rRNA gene of potential strains analyzed by BLAST from NCBI database.

## References

[1] Nutten S. (2015). Atopic dermatitis: Global epidemiology and risk factors. Ann. Nutr. Metab..

[2] Palmer C.N.A., Irvine A.D., Terron-Kwiatkowski A., Zhao Y., Liao H., Lee S.P., Goudie D.R., Sandilands A., Campbell L.E., Smith F.J.D. (2006). Common loss-of-function variants of the epidermal barrier protein filaggrin are a major predisposing factor for atopic dermatitis. Nat. Genet..

[3] Flohr C., Yeo L. (2011). Atopic Dermatitis and the Hygiene Hypothesis Revisited. Curr. Probl. Dermatol..

[4] Su C., Yang T., Wu Z., Zhong J., Huang Y., Huang T., Zheng E. (2017). Differentiation of T-helper cells in distinct phases of atopic dermatitis involves Th1/Th2 and Th17/Treg. Eur. J. Inflamm..

[5] Eichenfield L.F., Ahluwalia J., Waldman A., Borok J., Udkoff J., Boguniewicz M. (2017). Current guidelines for the evaluation and management of atopic dermatitis: A comparison of the Joint Task Force Practice Parameter and American Academy of Dermatology guidelines. J. Allergy Clin. Immunol..

[6] Lee S.Y., Lee E., Park Y.M., Hong S.J. (2018). Microbiome in the Gut-Skin Axis in Atopic Dermatitis. Allergy. Asthma Immunol. Res..

[7] Rather I.A., Bajpai V.K., Kumar S., Lim J., Paek W.K., Park Y.-H. (2016). Probiotics and Atopic Dermatitis: An Overview. Front. Microbiol..

[8] Fang Z., Li L., Zhang H., Zhao J., Lu W., Chen W. (2021). Gut Microbiota, Probiotics, and Their Interactions in Prevention and Treatment of Atopic Dermatitis: A Review. Front. Immunol..

[9] Majamaa H., Isolauri E. (1997). Probiotics: A novel approach in the management of food allergy. J. Allergy Clin. Immunol..

[10] Rosenfeldt V., Benfeldt E., Nielsen S.D., Michaelsen K.F., Jeppesen D.L., Valerius N.H., Paerregaard A. (2003). Effect of probiotic Lactobacillus strains in children with atopic dermatitis. J. Allergy Clin. Immunol..

[11] Choi C.-Y., Kim Y.-H., Oh S., Lee H.J., Kim J.H., Park S.H., Kim H.J., Lee S.J., Chun T. (2017). Anti-inflammatory potential of a heat-killed Lactobacillus strain isolated from Kimchi on house dust mite-induced atopic dermatitis in NC/Nga mice. J. Appl. Microbiol..

[12] Kothari D., Patel S., Kim S.-K. (2019). Probiotic supplements might not be universally-effective and safe: A review. Biomed. Pharmacother..

[13] Kim H.-J., Kim Y.-J., Kang M.-J., Seo J.-H., Kim H.-Y., Jeong S.K., Lee S.-H., Kim J.-M., Hong S.-J. (2012). A novel mouse model of atopic dermatitis with epicutaneous allergen sensitization and the effect of Lactobacillus rhamnosus. Exp. Dermatol..

[14] Jin H., He R., Oyoshi M., Geha R.S. (2009). Animal models of atopic dermatitis. J. Investig. Dermatol..

[15] Bumbacea R.S., Corcea S.L., Ali S., Dinica L.C., Fanfaret I.S., Boda D. (2020). Mite allergy and atopic dermatitis: Is there a clear link? (Review). Exp. Ther. Med..

[16] Smith P.D., Smythies L.E., Shen R., Greenwell-Wild T., Gliozzi M., Wahl S.M. (2011). Intestinal macrophages and response to microbial encroachment. Mucosal Immunol..

[17] Wang S., Ye Q., Zeng X., Qiao S. (2019). Functions of Macrophages in the Maintenance of Intestinal Homeostasis. J. Immunol. Res..

[18] Jorjão A.L., de Oliveira F.E., Leão M.V.P., Carvalho C.A.T., Jorge A.O.C., de Oliveira L.D. (2015). Live and Heat-Killed Lactobacillus rhamnosus ATCC 7469 May Induce Modulatory Cytokines Profiles on Macrophages RAW 264.7. Sci. World J..

[19] Hong W.-S., Chen H.-C., Chen Y.-P., Chen M.-J. (2009). Effects of kefir supernatant and lactic acid bacteria isolated from kefir grain on cytokine production by macrophage. Int. Dairy J..

[20] Iyer S.S., Cheng G. (2012). Role of interleukin 10 transcriptional regulation in inflammation and autoimmune disease. Crit. Rev. Immunol..

[21] Leung D.Y.M. (1993). Role of IgE in atopic dermatitis. Curr. Opin. Immunol..

[22] Wollenberg A., Thomsen S.F., Lacour J.-P., Jaumont X., Lazarewicz S. (2021). Targeting immunoglobulin E in atopic dermatitis: A review of the existing evidence. World Allergy Organ. J..

[23] Liu F.-T., Goodarzi H., Chen H.-Y. (2011). IgE, Mast Cells, and Eosinophils in Atopic Dermatitis. Clin. Rev. Allergy Immunol..

[24] Lee S.-H., Yoon J.-M., Kim Y.-H., Jeong D.-G., Park S., Kang D.-J. (2016). Therapeutic effect of tyndallized Lactobacillus rhamnosus IDCC 3201 on atopic dermatitis mediated by down-regulation of immunoglobulin E in NC/Nga mice. Microbiol. Immunol..

[25] Romagnani S. (2000). T-cell subsets (Th1 versus Th2). Ann. Allergy Asthma Immunol..

[26] Owens S., Howell M.D. (2019). Ruxolitinib Cream Suppresses Th2 Inflammation in Adult Patients With Atopic Dermatitis. J. Allergy Clin. Immunol..

[27] Kim J.-Y., Park B.-K., Park H.-J., Park Y.-H., Kim B.-O., Pyo S. (2013). Atopic dermatitis-mitigating effects of new Lactobacillus strain, Lactobacillus sakei probio 65 isolated from Kimchi. J. Appl. Microbiol..

[28] Kim H.W., Hong R., Choi E.Y., Yu K., Kim N., Hyeon J.Y., Cho K.K., Choi I.S., Yun C.-H. (2018). A Probiotic Mixture Regulates T Cell Balance and Reduces Atopic Dermatitis Symptoms in Mice. Front. Microbiol..

