# Supplementary material for: A Heat-Killed Probiotic Mixture Regulates Immune T Cells Balance and IgE Production in House Dust Mite Extraction-Induced Atopic Dermatitis Mice
Source: Microorganisms. 2022 Sep 21;10(10):1881. doi: 10.3390/microorganisms10101881 (PMC9607426; doi:10.3390/microorganisms10101881)
Supplement: Supplementary file 1 [file microorganisms-10-01881-s001.zip › microorganisms-1904008-supplementary.pdf]

A Heat-killed Probiotic Mixture Regulates Immune T cells Balance and IgE Production in House Dust Mite Extraction-induced Atopic Dermatitis Mice

Hsin-Yu Chen[1#], Yung-Tsung Chen[2#], Kuan-Yi Li[1], Hsiao-Wen Huang[1], Yu-Chun Lin[3], Ming-Ju Chen[1*]

[1]Department of Animal Science and Technology, National Taiwan University, Taipei City, Taiwan

[2]Department of Food Science, National Taiwan Ocean University, Keelung City, Taiwan

[3]Livestock Research Institute, Council of Agriculture, Executive Yuan, Tainan, Taiwan

[#]Hsin-Yun Chen and Yung-Tsung Chen contributed equally to this work.

***Corresponding author:**
Ming-Ju Chen
Department of Animal Science and Technology
National Taiwan University, Taipei City, Taiwan
No. 50, Ln. 155, Sec. 3, Keelung Rd., Da’an Dist., Taipei City 106032, Taiwan
email: cmj@ntu.edu.tw

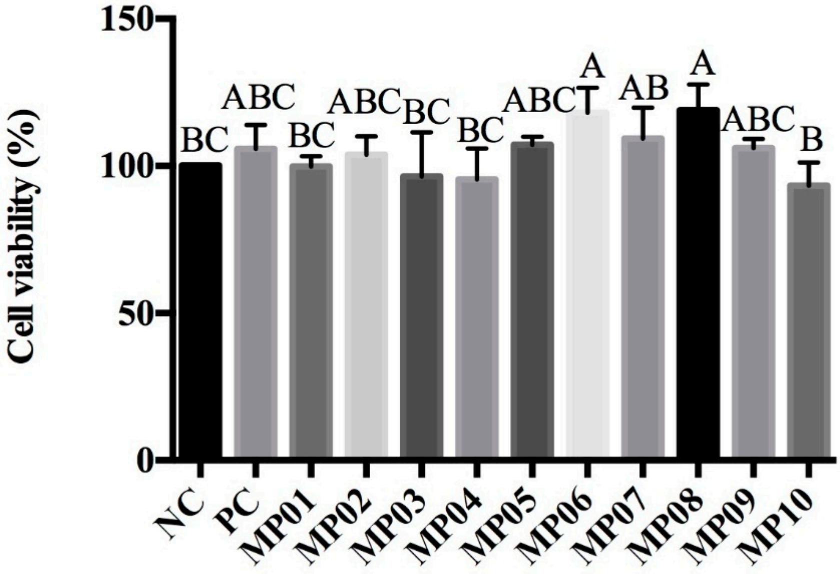

Supplementary Figure S1. Effects of the different LAB strains on the cell viability analyzed by MTT assay after 24-hour co-culturing with RAW 264.7. NC: Negative control; PC: Positive control (50 ng/mL LPS). Value represents means ± SD. (n=3). Data were analyzed by ANOVA with Duncan's multiple comparison tests. Means for each group without a common letter are significantly different ($P < 0.05$).

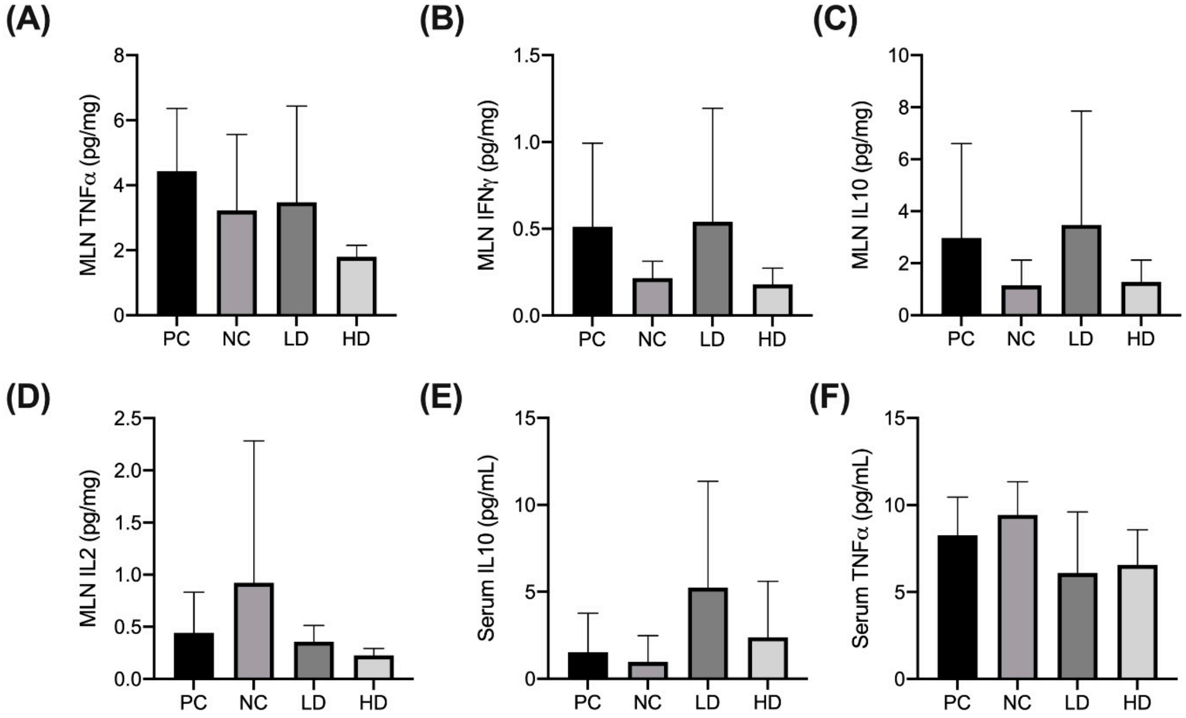

Supplementary Figure S2. Effects of a combination of heat-killed MP01 and MP02 strains on cytokines production in mesenteric lymph nodes (MLN) and serum of HDM-extraction induced AD mice. (A)TNF-α, (B)IFN-γ, (C)IL-10 and (D)IL-2 in mesenteric lymph nodes and (E)IL-10 and (F)TNF-α cytokines in serum of HDM-extraction induced AD mice after treatment. Data were analyzed by one-way ANOVA with Tukey's multiple comparison test. Bars indicate means ± SD.

Supplementary Table S1. 16S rRNA gene of potential strains analyzed by BLAST from NCBI database

Potential strains	Species	Identity	Species	Identity	Species	Identity
MP01	*Lactococcus lactis*	100%	*Lactococcus lactis*	100%	*Lactococcus lactis*	99.87%
MP02	*Lactobacillus paracasei*	99%	*Lactobacillus paracasei*	99%	*Lactobacillus zeae*	99%
